# Relationship between subjective fall risk assessment and falls and fall-related fractures in frail elderly people

**DOI:** 10.1186/1471-2318-11-40

**Published:** 2011-08-12

**Authors:** Hiroyuki Shimada, Megumi Suzukawa, Tatsuro Ishizaki, Kumiko Kobayashi, Hunkyung Kim, Takao Suzuki

**Affiliations:** 1 Section for Health Promotion, Department of Health and Medical Care, Center for Development of Advanced Medicine for Dementia, National Center for Geriatrics and Gerontology, Obu, Japan; 2 Faculty of Health Sciences, Department of Rehabilitation, Course of Physical Therapy, University of Human Arts and Science, Saitama, Japan; 3 Human Care Research Team, Tokyo Metropolitan Institute of Gerontology, Tokyo, Japan; 4 Tsukui Corporation, Yokohama, Japan; 5 Research Team for Promoting Independence of the Elderly, Tokyo Metropolitan Institute of Gerontology, Tokyo, Japan; 6 National Institute for Longevity Sciences, National Center for Geriatrics and Gerontology, Obu, Japan

## Abstract

**Background:**

Objective measurements can be used to identify people with risks of falls, but many frail elderly adults cannot complete physical performance tests. The study examined the relationship between a subjective risk rating of specific tasks (SRRST) to screen for fall risks and falls and fall-related fractures in frail elderly people.

**Methods:**

The SRRST was investigated in 5,062 individuals aged 65 years or older who were utilized day-care services. The SRRST comprised 7 dichotomous questions to screen for fall risks during movements and behaviours such as walking, transferring, and wandering. The history of falls and fall-related fractures during the previous year was reported by participants or determined from an interview with the participant's family and care staff.

**Results:**

All SRRST items showed significant differences between the participants with and without falls and fall-related fractures. In multiple logistic regression analysis adjusted for age, sex, diseases, and behavioural variables, the SRRST score was independently associated with history of falls and fractures. Odds ratios for those in the high-risk SRRST group (≥ 5 points) compared with the no risk SRRST group (0 point) were 6.15 (p < 0.01) for a single fall, 15.04 (p < 0.01) for recurrent falls, and 5.05 (p < 0.01) for fall-related fractures. The results remained essentially unchanged in subgroup analysis accounting for locomotion status.

**Conclusion:**

These results suggest that subjective ratings by care staff can be utilized to determine the risks of falls and fall-related fractures in the frail elderly, however, these preliminary results require confirmation in further prospective research.

## Background

Falls and fall-related fractures are a common cause of disability in elderly people [1], and preventing falls is an urgent medical and social issue. Numerous studies have identified factors that predict an increased risk of falls, and many validated assessment tools have been developed to determine fall risks for elderly people [2,3]. Although falls can be caused by multiple factors, mobility impairments such as gait and balance disorders are among the most common predisposing conditions [4,5].

Our previous studies have identified the best mobility tests [6] and a physical performance test [7] for predicting falls in the elderly. These objective measurements can be used to identify people who are appropriate for and who will gain benefit from targeted falls prevention interventions. However, we found that about half of the frail elderly subjects could not complete physical performance tests such as the functional reach test and tandem walk test [7]. In addition, cognitive impairment, particularly confusion, impaired orientation, and misperception of functional ability, is one of the most important risk factors for falls in elderly people [8,9] and is likely to be an important inclusion in a screening tool. Successful strategies for preventing falls in frail elderly people with cognitive impairments are yet to be identified conclusively [10] and appropriate screening tools for these individuals are needed.

Some subjective assessments by care staff have been developed for identifying fall risks in frail elderly adults [11-13]. In a residential facility, staff members possess knowledge of their residents' potential fall risk over a 24-hour period, and this encompasses both predisposing and precipitating factors. Therefore, their global assessment of fall risk could have the highest predictive validity in relation to falls [13]. These global assessment scales are composed of one item, e.g. 'how do you judge the risk that Mr or Mrs × will fall within 6 months--high or low?' [11,13], which can be used easily in clinical settings, but a global assessment cannot identify specific fall risks and appropriate interventions in frail elderly persons who have multifactorial risks for falls. We determined seven specific tasks with high risks of falls based on a nationwide survey of falls in the frail elderly [7,14], and identified the relationship between these tasks and falls in our preliminary study [15]. However, it was not clear that these tasks were related independently with falls and fall-related fractures in a large population study.

The purpose of this study was to develop the subjective risk rating of specific tasks (SRRST) for screening for the risk of falls and fall-related fractures. Subjects were frail elderly people enrolled in the Tsukui Ordered Useful Care for Health (TOUCH) program which provides day-care services.

## Methods

### Participants

This study recruited 5,062 elderly participants (mean age, 82.6 ± 7.4 years) enrolled in the TOUCH program. To enrol in TOUCH, an individual must be aged 65 or older and have been certified as needing long-term care by the Japanese public long-term care insurance system [16]. The TOUCH sites are located throughout Japan and provide comprehensive, facility-based day-care services. TOUCH clients have some physical disability and frailty, as defined by the presence of weakness, reduced physical activity or slow gait, which is in accordance with the widely accepted definition of frailty [17]. We limited the participants of this study to those who were aged less than 65 years and who had missing value in measurements. Informed consent was obtained from all participants or a family member prior to their inclusion in the study and the Ethics Committee of the Tokyo Metropolitan Institute of Gerontology approved the study protocol.

### Study procedures

This study was performed by cross-sectional design and falls and fall-related fractures were investigated retrospectively for a one-year period. Prior to the commencement of the study, all staff received a measurement manual which mentioned the correct protocols for administering all of the assessment measures included in the study.

### Falls and fall-related fractures during the previous year

A fall was defined as "an event that resulted in a person coming to rest unintentionally on the ground or another lower level that did not result from a major intrinsic event or an overwhelming hazard" [18,19]. Falls and fall-related fractures were measured retrospectively for a one-year period via a self-report questionnaire and care records. A caregiver or family member provided information on the participant's annual incidence of falls and fall-related fractures when the trained nurses or care workers recognized that a participant had problems recalling such events.

### Subjective risk rating of specific tasks (SRRST)

The SRRST was conducted by day-centre staff who had nursing, allied health or similar qualifications, and they were familiar with their clients. The staff answered the questions of the SRRST based on the present status of the participants. The SRRST consisted of the following items: 1) "Do you feel there is a risk of falls when the client (Mr or Mrs X) is walking?"; 2) "Do you feel there is a risk of falls when the client is transferring in bed room, toilet, or bath room?"; 3) "Do you feel there is a risk of falls when the client is toileting?"; 4) "Do you feel there is a risk of falls when the client is ascending or descending stairs?"; 5) "Do you feel there is a risk of falls when the client is wandering?"; 6) "Do you feel there is risk of falls because the client exhibits risky behavior?"; 7) "Do you feel there is a risk of falls because the client is agitated?". The response to each item in the SRRST was designated as "yes" (1 point) or "no or not applicable" (0 points) [15]. The information of the SRRST and history of falls was obtained at the same time. Prior to the commencement of the study, three raters completed the SRRST twice at weekly intervals (n = 4 × 2 × 30), and test-retest and inter-rater (one physical therapist, one nurse, and two caregivers) reliability comparisons of total scores revealed intraclass correlation coefficients (ICCs) of 0.84 to 0.96 and 0.81, respectively [20].

### Potential confounding factors of falls

With reference to previous studies [2,21-24], we selected two demographic variables, eight primary diseases or general health statuses, and two behavioural variables as possible confounding factors of falls (Table 1). The demographic variables were sex and age. Primary diseases or general health status were recorded by the care staff, who identified the chronic condition from care records or symptoms. The following diseases and general health status were included in the analysis: history of stroke with symptoms of hemiparesis, knee osteoarthritis with pain, Parkinson disease, dementia, poor vision, urinary incontinence or frequency, psychotropic use, and walking aid use. Absence of habitual exercise and daily use of slippers or sandals were investigated as behavioural variables.

**Table 1:** Number of participants with falls and fall-related fractures and odds ratios of potential risk factors

	Single fall		Recurrent falls		Fractures	
	Number (%)	Odds ratio (95% CI)	Number (%)	Odds ratio (95% CI)	Number (%)	Odds ratio (95% CI)
Subjective risk rating of specific tasks						

Risk of falls during walking, yes	1068 (41.5)^†^	2.21 (2.01-2.43)	633 (24.6)^†^	3.15 (2.71-3.66)	123 (4.8)^†^	1.83 (1.36-2.46)
Risk of falls during transferring, yes	823 (41.7)^†^	1.80 (1.66-1.96)	504 (25.5)^†^	2.43 (2.14-2.76)	103 (5.2)^†^	1.89 (1.43-2.51)
Risk of falls during toileting, yes	568 (42.9)^†^	1.66 (1.53-1.80)	361 (27.3)^†^	2.18 (1.93-2.47)	65 (4.9)^†^	1.49 (1.11-2.00)
Risk of falls during stair ascending/descending, yes	1140 (39.2)^†^	2.13 (1.93-2.36)	685 (23.6)^†^	3.55 (2.99-4.22)	139 (4.8)^†^	2.10 (1.53-2.90)
Risk of falls during wandering, yes	453 (44.9)^†^	1.68 (1.55-1.83)	2.89 (28.7)^†^	2.16 (1.90-2.44)	66 (6.5)^†^	2.18 (1.63-2.91)
Risk of falls because of risky behaviors, yes	672 (41.6)^†^	1.66 (1.53-1.80)	424 (26.3)^†^	2.24 (1.98-2.54)	79 (4.9)^†^	1.55 (1.17-2.06)
Risk of falls because of agitation, yes	479 (45.0)^†^	1.70 (1.57-1.85)	316 (29.7)^†^	2.32 (2.05-2.62)	55 (5.2)^†^	1.55 (1.14-2.11)

Potential confounding factors						

Age, years^‡^ Falls or fractures	82.9 ± 7.5		83.0 ± 7.5		84.3 ± 6.9^†^	
No falls or fractures	82.5 ± 7.4		82.6 ± 7.4		82.6 ± 7.4	
Sex, female	1062 (30.1)	0.97 (0.89-1.06)	560 (15.8)	0.90 (0.79-1.03)	151 (4.3)^†^	1.77 (1.24-2.52)
Stroke, yes	345 (32.0)	1.07 (0.97-1.18)	175 (16.2)	0.99 (0.85-1.16)	41 (3.8)	1.03 (0.74-1.45)
Knee osteoarthritis and pain, yes	659 (36.7)^†^	1.36 (1.26-1.48)	362 (20.1)^†^	1.41 (1.25-1.60)	77 (4.3)	1.26 (0.95-1.67)
Dementia, yes	670 (34.3)^†^	1.23 (1.13-1.34)	387 (19.8)^†^	1.40 (1.23-1.58)	80 (4.1)	1.18 (0.89-1.57)
Poor vision, yes	239 (37.9)^†^	1.30 (1.16-1.45)	131 (20.8)*	1.32 (1.12-1.56)	26 (4.1)	1.13 (0.74-1.73)
Parkinson disease, yes	163 (44.7)^†^	1.53 (1.35-1.73)	104 (28.5)^†^	1.85 (1.55-2.20)	16 (4.4)	1.20 (0.73-1.98)
Use of psychotropics, yes	525 (37.0)^†^	1.33 (1.22-1.45)	283 (19.9)^†^	1.33 (1.17-1.52)	57 (4.0)	1.12 (0.82-1.51)
Urinary incontinence or frequency, yes	702 (36.2)^†^	1.35 (1.25-1.47)	403 (20.8)^†^	1.53 (1.35-1.73)	82 (4.2)	1.24 (0.94-1.65)
Absence of habitual exercise, yes	975 (33.7)^†^	1.31 (1.20-1.43)	561 (19.4)^†^	1.58 (1.38-1.81)	110 (3.8)	1.06 (0.80-1.41)
Use of slippers or sandals, yes	415 (36.3)^†^	1.27 (1.16-1.39)	185 (16.2)	0.99 (0.85-1.14)	63 (5.5)^†^	1.73 (1.28-2.32)
Use of walking aid, yes	887 (36.7)^†^	1.49 (1.37-1.63)	492 (20.3)^†^	1.60 (1.41-1.82)	109 (4.5)^†^	1.51 (1.14-2.01)

*p < .05, ^†^p < .01, ^‡^Mean ± standard deviation

### Statistical analysis

Each SRRST item and potential confounding factor was compared between the participants with and without a single fall, recurrent falls, and fall-related fractures using *t*-tests for age and chi-square tests for categorical variables. Odds ratios (ORs) of potential risk factors were also calculated for categorical variables.

Multiple logistic regression analysis was performed to explore the independent associations between total SRRST score and falls and fall-related fractures with potential confounding factors. Multiple logistic regression models included total SRRST score as an independent variable, which was categorized into no risk (0 point), low risk (1 to 2 points), moderate risk (3 to 4 points), and high risk (≥ 5 points). The SRRST categories were assessed by their P-values for trend and were used to calculate the OR and 95% confidence interval (95% CI) relative to the category of 'no risk' for each higher category. Covariates were added sequentially to the logistic model to evaluate the associations at different levels of adjustment. Model 1 included the SRRST category plus age and sex, and model 2 included the model 1 variables plus other possible confounding factors. The participants were divided into dependent walking and independent walking groups for subgroup analysis. Logistic regression analysis (model 2) was performed in each group. The validity of the model was quantified using the C-Index and Hosmer-Lemeshow statistic for goodness of fit. Sensitivity and specificity statistics were used to determine the ability of classification in the SRRST. Sensitivity and specificity for falls and fall-related fractures were calculated in each SRRST score. Cut-points for maximizing the sensitivity and specificity for each score were determined using the closest-to-(0, 1) criterion [25]. All data management and statistical computations were performed using the SPSS 17.0 software package (SPSS Inc., Chicago, IL, USA).

## Results

The participants were recruited from 88 TOUCH demonstration sites (35% of all sites) and completed the investigation. About 65% of the TOUCH sites (about 19,800 elderly people) could not complete the investigation. Table 2 shows the characteristics of the participants (Table 2).

**Table 2:** Characteristics (number and percent) of the participants (n = 5,062)

Age*	83 (41)
Women	3,541 (70.0)
Single fall during a one-year period	1,536 (30.3)
Recurrent falls during a one-year period	828 (16.4)
Fall-related fracture during a one-year period	188 (3.7)
Femoral fracture	74 (1.5)
Fracture of the skull, trunk, pelvic, and lower legs	68 (1.3)
Fracture of the arms	46 (0.9)
Stroke	1,077 (21.3)
Knee osteoarthritis with pain	1,798 (35.5)
Dementia	1,953 (38.6)
Poor vision	630 (12.4)
Parkinson disease	365 (7.2)
Use of psychotropics	1,420 (28.1)
Urinary incontinence or frequency	1,941 (38.3)
Absence of habitual exercise	2,889 (57.1)
Use of slippers and sandals	1,144 (22.6)
Use of a walking aid	2,418 (47.8)
Mobility status	
Independent gait	2,930 (57.9)
Independent transfers	953 (18.8)
Independent sit up	589 (11.6)
Dependent sit up	590 (11.7)

* median (range)

### Number of participants with falls and fall-related fractures

Of the 5,062 elderly people, 1536 (30.3%) reported a single fall in the previous year, 828 (16.4%) had recurrent falls, and 188 (3.7%) experienced fall-related fractures. Of the participants with fractures, 74 (39.4%) had a femoral fracture, 68 (36.2%) participants had a fracture of the skull, trunk, pelvic, or lower leg, and 46 (24.5%) experienced a fracture of the arm.

### Comparison between participants with and without falls and fall-related fractures

All SRRST items showed significant differences between those with and without a fall, recurrent falls, and fall-related fractures. In terms of potential confounding variables, there were significant differences for all except history of stroke when single fallers and non fallers were compared. When recurrent fallers were compared with non-recurrent fallers there was a significant difference for all potential confounders except for history of stroke and daily use of slippers or sandals. Compared with participants without fractures, those with fractures were significantly more likely to report daily use of slippers or sandals or use of walking aids (Table 1).

Among the SRRST items, ORs of the participants with risk to those without risk were 1.66 to 2.21 for a single fall, 2.16 to 3.55 for recurrent falls, and 1.49 to 2.18 for fall-related fractures. The ORs of significant confounders were 1.23 to 1.53 for a single fall, 1.32 to 1.85 for recurrent falls, and 1.51 to 1.73 for fall-related fractures. The highest ORs for a single fall, recurrent falls, and fall-related fractures were recognized for the SRRST items of risk of falls during walking, stair ascending/descending, and wandering, respectively (Table 1).

### Risk factors for falls

**Figure 1:**
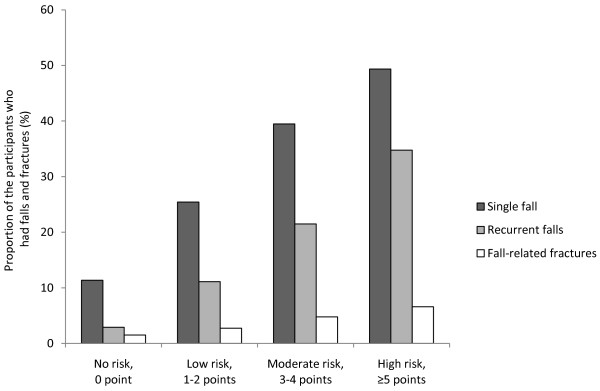
**Proportion of the participants who had single fall, recurrent falls, and **fall-related **fractures according to fall risk**. Frail, elderly participants were categorized into four **fall risk **groups by SRRST score. The rate of single fall, recurrent falls, and fall-related fractures increased in accordance with the risk of falls based on the SRRST score.

**Figure 2:**
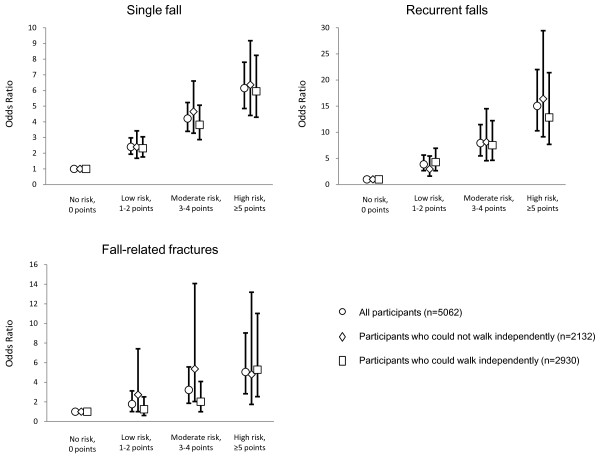
**Odds ratios and 95% confidence intervals of SRRST categories for a single fall, recurrent falls, and fall-related fractures**. Models were adjusted for all confounding factors (model 2). The participants were divided into dependent-walking and independent-walking groups.

The multiple logistic regression models showed significant relationships between falls and fall-related fractures and SRRST categories (Table 3). Participants who had higher fall risk on the SRRST had higher rates of falling and fall-related fractures (Figure 1). In model 1, which adjusted for age and sex, the OR for a single fall, recurrent falls, and fall-related fractures increased as the SRRST score increased, and P for trend of all models showed significance. The ORs of the high-risk group compared with the no-risk group were 7.56 (95% confidence interval (95% CI); 6.07-9.42) for single fall; 17.71 (95% CI; 12.32-25.45) for recurrent falls, and 4.65 (95% CI; 2.73-7.94) for fall-related fractures (P for trend < 0.01). The results remained essentially unchanged after controlling for other confounders (Table 3, model 2). The highest ORs of factors related to single fall, recurrent falls, and fall-related fractures were for the high-risk group in the SRRST in all logistic models. The p-values of the Hosmer-Lemeshow statistics were > 0.05 in both logistic models (p = 0.12-0.72) and the C-index showed moderate model-fit in nearly all cases (0.67-0.74). In the subgroup analysis, the significant odds ratios remained essentially the same in the dependent walking and independent walking groups, with the exception of fall-related fracture. Regarding fall-related fractures in the dependent walking group, there were no significant odds ratios when the low and moderate risk groups were compared to the no-risk group of the SRRST (Figure 2).

**Table 3:** Odds ratios for falls and fall-related fractures by SRRST category and confounders

	Single fall		Recurrent falls		Fractures	
	Model 1 Odds ratio (95% CI)	Model 2 Odds ratio (95% CI)	Model 1 Odds ratio (95% CI)	Model 2 Odds ratio (95% CI)	Model 1 Odds ratio (95% CI)	Model 2 Odds ratio (95% CI)
**Subjective risk rating of specific tasks**						

No risk, 0 points	1.00^‡^	1.00^‡^	1.00^‡^	1.00^‡^	1.00^‡^	1.00^‡^
Low risk, 1-2 points	2.65 (2.14-3.28)^†^	2.40 (1.94-2.98)^†^	4.17 (2.88-6.06)^†^	3.88 (2.67-5.64)^†^	1.80 (1.03-3.15)*	1.77 (1.01-3.12)*
Moderate risk, 3-4 points	5.06 (4.11-6.23)^†^	4.21 (3.39-5.23)^†^	9.11 (6.36-13.05)^†^	7.94 (5.5-11.47)^†^	3.24 (1.91-5.48)^†^	3.22 (1.86-5.57)^†^
High risk, ≥ 5 points	7.56 (6.07-9.42)^†^	6.15 (4.85-7.8)^†^	17.71 (12.32-25.45)^†^	15.04 (10.29-22)^†^	4.65 (2.73-7.94)^†^	5.05 (2.83-9.03)^†^
P for trend	< 0.01	< 0.01	< 0.01	< 0.01	< 0.01	< 0.01

**Potential confounding factors**						

Age, years	1.01 (1.00-1.01)	1.01 (1.00-1.02)	1.01 (0.99-1.02)	1.00 (0.99-1.02)	1.02 (1.00-1.05)*	1.03 (1.01-1.05)*
Sex, female	0.97 (0.84-1.11)	0.94 (0.81-1.09)	0.91 (0.77-1.08)	0.89 (0.74-1.06)	1.72 (1.19-2.50)^†^	1.76 (1.20-2.58)^†^
History of stroke with symptoms of hemiparesis		1.04 (0.88-1.22)		0.88 (0.72-1.08)		1.32 (0.91-1.93)
Knee osteoarthritis with pain		1.28 (1.12-1.47)^†^		1.31 (1.11-1.54)^†^		1.00 (0.73-1.37)
Parkinson disease		1.44 (1.14-1.81)^†^		1.51 (1.16-1.96)^†^		1.10 (0.64-1.88)
Dementia		0.93 (0.81-1.08)		0.95 (0.80-1.14)		0.87 (0.62-1.22)
Poor vision		1.05 (0.87-1.27)		1.00 (0.80-1.25)		0.87 (0.56-1.34)
Urinary incontinence or frequency		1.09 (0.95-1.26)		1.06 (0.89-1.26)		0.96 (0.69-1.34)
Use of psychotropics		1.22 (1.06-1.40)^†^		1.07 (0.90-1.28)		0.95 (0.68-1.32)
Use of walking aid		1.20 (1.05-1.38)^†^		1.14 (0.96-1.35)		1.07 (0.78-1.47)
Absence of habitual exercise		1.04 (0.90-1.19)		1.15 (0.96-1.37)		0.81 (0.59-1.12)
Daily use of slippers or sandals		1.23 (1.06-1.43)^†^		0.81 (0.67-0.98)*		1.67 (1.21-2.30)^†^
						
C-index, value (95% CI)	0.68 (0.66-0.70)^†^	0.70 (0.68-0.71)^†^	0.72 (0.71-0.74)^†^	0.74 (0.72-0.75)^†^	0.67 (0.63-0.71)^†^	0.69 (0.65-0.73)^†^
Hosmer-Lemeshow test, p value	0.51	0.48	0.72	0.57	0.72	0.12

*p < .05, ^†^p < .01

^‡^Odds ratios in the SRRST category were calculated in the low, moderate, and high risk relative to the no risk.

### Sensitivity and specificity

Table 4 shows the sensitivity and specificity of each SRRST score for falls and fall-related fractures. Cut-points for maximizing the sensitivity and specificity were 2/3 point in all of a single fall, recurrent falls and fall-related fractures. Sensitivity and specificity of 2/3 cut-point in a single fall, recurrent falls and fall-related fractures were 0.66 and 0.63, 0.75 and 0.60, and 0.68 and 0.55, respectively.

**Table 4:** Sensitivity and specificity of SRRST scores for falls and fall-related fractures

SRRST score	Single fall		Recurrent falls		Fractures	
	
	Sensitivity (95% CI)	Specificity (95% CI)	Sensitivity (95% CI)	Specificity (95% CI)	Sensitivity (95% CI)	Specificity (95% CI)
0/1 point	0.91 (0.90 to 0.92)	0.30 (0.29 to 0.32)	0.96 (0.94 to 0.97)	0.28 (0.26 to 0.29)	0.90 (0.85 to 0.94)	0.24 (0.23 to 0.26)
1/2 point	0.80 (0.78 to 0.82)	0.47 (0.45 to 0.49)	0.87 (0.85 to 0.90)	0.44 (0.42 to 0.45)	0.79 (0.72 to 0.84)	0.39 (0.38 to 0.41)
2/3 point	0.66 (0.63 to 0.68)	0.63 (0.61 to 0.64)	0.75 (0.72 to 0.78)	0.60 (0.58 to 0.61)	0.68 (0.61 to 0.74)	0.55 (0.53 to 0.56)
3/4 point	0.48 (0.46 to 0.51)	0.76 (0.75 to 0.78)	0.58 (0.55 to 0.62)	0.74 (0.73 to 0.76)	0.50 (0.43 to 0.57)	0.70 (0.68 to 0.71)
4/5 point	0.30 (0.28 to 0.32)	0.87 (0.86 to 0.88)	0.39 (0.36 to 0.42)	0.86 (0.85 to 0.87)	0.32 (0.26 to 0.39)	0.82 (0.81 to 0.83)
5/6 point	0.14 (0.13 to 0.16)	0.94 (0.93 to 0.95)	0.22 (0.20 to 0.25)	0.93 (0.92 to 0.94)	0.13 (0.09 to 0.19)	0.91 (0.90 to 0.92)
6/7 point	0.07 (0.06 to 0.09)	0.97 (0.97 to 0.98)	0.10 (0.08 to 0.12)	0.97 (0.96 to 0.97)	0.02 (0.01 to 0.05)	0.96 (0.95 to 0.96)

## Discussion

There are many distinct and multifactorial causes for falls in elderly people, including low muscle strength, balance and gait disturbances, cognitive function decline, environmental hazards, and low or high activity levels. Objective measures such as physical tests can provide accurate information in accordance with the task tested, but predictive validity of these tests are inadequate in frail elderly people with multiple risks of falls. This may be explained by the multifactorial nature of falls, which makes the notion of a single screening tool with excellent predictive accuracy an unrealistic goal. Nordin (2008) reported that staff members' assessment of their residents' fall risk as well as history of previous falls appeared superior to performance-based measures of falls in frail elderly people [13]. We therefore examined the utility of an objective assessment tool to identify useful measures for screening frail elderly people for fall risk.

In the comparative analysis, when compared with non-fallers, participants who had experienced falling were more likely to have a fall risk (with the exception of history of stroke and use of slippers and sandals). In contrast, when compared with those without fall-related fractures, participants who had fall-related fractures did not show significant differences in many potential confounding factors, although all SRRST items showed significant differences. These results suggest that the subjective assessment used in the SRRST was useful to examine the risk of fractures in the frail elderly.

Multiple regression models revealed that the SRRST score was associated with falling as well as fall-related fracture, even when adjusted for many confounding factors. Odds ratios were markedly higher for recurrent falls than for single fall and fall-related fractures. A previous study suggested that infrequent or isolated falls are more unpredictable events than multiple falls and less likely to result from underlying neurologic or musculoskeletal problems [18]. The incidence of fall-related fractures is also influenced by low bone density which was not measured in this study [26-28]. These factors may have weakened the relationships between the SRRST and a single fall and fall-related fractures. Higher odds ratios, however, remained between the SRRST and history of falling and fractures than previously reported odds ratios calculated from the cut-off points of objective performance tests in frail elderly people who participated in the TOUCH [7]. Cut-points for maximizing the sensitivity and specificity were 2/3 point in all of a single fall, recurrent falls and fall-related fractures. Care providers may require attention to risk of falls and fall-related fractures in the frail elderly adults who have a score 3 points and over in the SRRST.

Why did staff assessments show close relationships with falls and fall-related fractures? Falling is induced by multidimensional factors, and the primary cause of falling may vary among frail elderly adults who have many risk factors for falls. Thus, it is difficult to determine the primary risks for falls in all frail elderly adults using objective measures that can identify only specific issues. In contrast, subjective evaluations can determine combined risks of falling based on various information such as physical functions, daily activity status, and risky behaviors, although these evaluations cannot give clear, specific and objective risks for falling. The combined information is important for identifying risks of falls and preventing falls in frail elderly people, because correct risk-assessments by care staff may lead to successful assessment and interventions for preventing falls [29,30]. We reported previously that an intervention study using supervision technique based on the assessment of fall-risk behaviors can reduce the risk of falling in institutionalized elderly people [31]. Thus, we considered that the assessment and intervention used in the SRRST may be useful for preventing falls in frail elderly people. Furthermore, the SRRST has the strength of being designed for frail elderly people. Although risk factors for falls differ between elderly adults who can and cannot stand unaided [32], nearly all risks identified by the SRRST showed significant odds ratios for falls and fall-related fractures in the dependent walking and independent walking groups. Future research should include a prospective measurement of falls in order to more accurately determine the validity of the SRRST for this population and perform an intervention study to reveal the effects of the SRRST on intervention.

One of the limitations of our study is that we performed a cross-sectional study and analysed retrospectively recalled falls. This is known to be a less accurate measure than prospectively recalled falls [33]. It is possible that underreporting of falls by participants may have led an underestimation of the rates of falls. Therefore, further investigation of the validity of these tests in predicting falls in frail elderly people using a prospective study design is recommended. Second, the investigations of the SRRST and history of falls were investigated at the same time. Thus, the information of the history of falls might affect subjective judgments of the testers. However, correct judgments of the SRRST may require multidimensional information included the history of falls in the elderly adults and testers, i.e. care providers, may know history of falls of their clients through daily care. In other words, testers who had information of falls history in the subjects could measure correctly the risk of falls using the SRRST.

## Conclusion

In conclusion, this study developed the SRRST as a subjective assessment for identifying risk of falls in the frail elderly people. Numerous studies developed fall risk assessment tools which evaluate using objective physical or cognitive measurements [2]. Unfortunately, some frail elderly adults cannot perform objective assessments to screen fall risks although these assessment tools may judge almost frail elderly as high risk individuals and identify multiple risks for falling [7]. The SRRST can evaluate easily the specific fall risks and have high feasibility in the elderly. This study provides the evidence that subjective assessment by staff was associated with risk of falling and fall-related fractures in frail elderly people. We encourage providing a fall prevention strategy to the frail elderly who had some risks for falls in your subjective judgments. Future research need to determine the predictive validity of incidence of falls and fractures in the frail elderly people.

## Abbreviations

SRRST: Subjective Risk Rating of Specific Tasks; TOUCH: Tsukui Ordered Useful Care for Health; ICC: intraclass correlation coefficient; OR: Odds Ratio; 95% CI: 95% confidence interval.

## Competing interests

The authors declare that they have no competing interests.

## Authors' contributions

HS and MS were responsible for the study concept and design. HS was responsible for the draft of the manuscript. MI, TI, KH, and TS were responsible for the critical revision of the manuscript for important intellectual content. KK was responsible for the coordination of acquisition of data. All authors were responsible for the final approval of the manuscript.

## Pre-publication history

The pre-publication history for this paper can be accessed here:

http://www.biomedcentral.com/1471-2318/11/40/prepub

## References

[B1] Ministry of Health, Labor and Welfare National nutrition survey in Japan 2002 2002 Tokyo: Ministry of Health, Labor and Welfare 10.2169/internalmedicine.41.70 11838604

[B2] Lord SR Sherrington C Menz HB Close JCT Falls in older people: risk factors and strategies for prevention 2007 2 Cambridge: Cambridge University Press

[B3] Scott V Votova K Scanlan A Close J Multifactorial and functional mobility assessment tools for fall risk among older adults in community, home-support, long-term and acute care settings Age Ageing 2007 36 2 130 139 10.1093/ageing/afl165 17293604

[B4] Robbins AS Rubenstein LZ Josephson KR Schulman BL Osterweil D Fine G Predictors of falls among elderly people. Results of two population-based studies Arch Intern Med 1989 149 7 1628 1633 10.1001/archinte.149.7.1628 2742437

[B5] Lord SR Menz HB Tiedemann A A physiological profile approach to falls risk assessment and prevention Phys Ther 2003 83 3 237 252 12620088

[B6] Tiedemann A Shimada H Sherrington C Murray S Lord S The comparative ability of eight functional mobility tests for predicting falls in community-dwelling older people Age Ageing 2008 37 4 430 435 10.1093/ageing/afn100 18487264

[B7] Shimada H Suzukawa M Tiedemann A Kobayashi K Yoshida H Suzuki T Which neuromuscular or cognitive test is the optimal screening tool to predict falls in frail community-dwelling older people? Gerontology 2009 55 5 532 538 10.1159/000236364 19776609

[B8] Salgado R Lord SR Packer J Ehrlich F Factors associated with falling in elderly hospital patients Gerontology 1994 40 6 325 331 10.1159/000213607 7867963

[B9] Stevenson B Mills EM Welin L Beal KG Falls risk factors in an acute-care setting: a retrospective study Can J Nurs Res 1998 30 1 97 111 9726185

[B10] Jensen J Nyberg L Gustafson Y Lundin-Olsson L Fall and injury prevention in residential care--effects in residents with higher and lower levels of cognition J Am Geriatr Soc 2003 51 5 627 635 10.1034/j.1600-0579.2003.00206.x 12752837

[B11] Lundin-Olsson L Jensen J Nyberg L Gustafson Y Predicting falls in residential care by a risk assessment tool, staff judgement, and history of falls Aging Clin Exp Res 2003 15 1 51 59 12841419 10.1007/BF03324480

[B12] Izumi K Makimoto K Kato M Hiramatsu T Prospective study of fall risk assessment among institutionalized elderly in Japan Nurs Health Sci 2002 4 4 141 147 10.1046/j.1442-2018.2002.00119.x 12406200

[B13] Nordin E Lindelöf N Rosendahl E Jensen J Lundin-Olsson L Prognostic validity of the Timed Up-and-Go test, a modified Get-Up-and-Go test, staff's global judgement and fall history in evaluating fall risk in residential care facilities Age Ageing 2008 37 4 442 448 10.1093/ageing/afn101 18515291

[B14] Suzukawa M Shimada H Makizako H Watanabe S Suzuki T [Incidence of falls and fractures in disabled elderly people utilizing long-term care insurance] Nippon Ronen Igakkai zasshi 2009 46 4 334 340 10.3143/geriatrics.46.334 19713666

[B15] Suzukawa M Shimada H Tamura M Suzuki T Inoue N The relationship between the subjective risk rating of specific tasks and falls in frail elderly people J Phys Ther Sci 2011 23 3 425 429 10.1589/jpts.23.425

[B16] Tsutsui T Muramatsu N Japan's universal long-term care system reform of 2005: containing costs and realizing a vision J Am Geriatr Soc 2007 55 9 1458 1463 10.1111/j.1532-5415.2007.01281.x 17767690

[B17] Fried LP Ettinger WH Lind B Newman AB Gardin J Physical disability in older adults: a physiological approach. Cardiovascular Health Study Research Group J Clin Epidemiol 1994 47 7 747 760 10.1016/0895-4356(94)90172-4 7722588

[B18] Nevitt MC Cummings SR Kidd S Black D Risk factors for recurrent nonsyncopal falls. A prospective study JAMA 1989 261 18 2663 2668 10.1001/jama.261.18.2663 2709546

[B19] Cumming RG Sherrington C Lord SR Simpson JM Vogler C Cameron ID Naganathan V Prevention of Older People's Injury Falls Prevention in Hospitals Research Group Cluster randomised trial of a targeted multifactorial intervention to prevent falls among older people in hospital BMJ 2008 336 7647 758 760 10.1136/bmj.39499.546030.BE 18332052 PMC2287238

[B20] Hashidate H Shimada H Shiomi T Sasamoto N Usefulness of the subjective risk rating of specific tasks for falling in frail older people J Phys Ther Sci 2011 23 3 519 524 10.1589/jpts.23.519

[B21] Rubenstein LZ Josephson KR The epidemiology of falls and syncope Clin Geriatr Med 2002 18 2 141 158 10.1016/S0749-0690(02)00002-2 12180240

[B22] Tinetti ME Speechley M Ginter SF Risk factors for falls among elderly persons living in the community N Engl J Med 1988 319 26 1701 1707 10.1056/NEJM198812293192604 3205267

[B23] Fleming BE Pendergast DR Physical condition, activity pattern, and environment as factors in falls by adult care facility residents Arch Phys Med Rehabil 1993 74 6 627 630 10.1016/0003-9993(93)90161-3 8503753

[B24] Kiely DK Kiel DP Burrows AB Lipsitz LA Identifying nursing home residents at risk for falling J Am Geriatr Soc 1998 46 5 551 555 9588366 10.1111/j.1532-5415.1998.tb01069.x

[B25] Perkins NJ Schisterman EF The inconsistency of "optimal" cutpoints obtained using two criteria based on the receiver operating characteristic curve Am J Epidemiol 2006 163 7 670 675 10.1093/aje/kwj063 16410346 PMC1444894

[B26] Dargent-Molina P Favier F Grandjean H Baudoin C Schott AM Hausherr E Meunier PJ Bréart G Fall-related factors and risk of hip fracture: the EPIDOS prospective study Lancet 1996 348 9021 145 149 10.1016/S0140-6736(96)01440-7 8684153

[B27] Cummings SR Nevitt MC Browner WS Stone K Fox KM Ensrud KE Cauley J Black D Vogt TM Risk factors for hip fracture in white women. Study of Osteoporotic Fractures Research Group N Engl J Med 1995 332 12 767 773 10.1056/NEJM199503233321202 7862179

[B28] Chandler JM Zimmerman SI Girman CJ Martin AR Hawkes W Hebel JR Sloane PD Holder L Magaziner J Low bone mineral density and risk of fracture in white female nursing home residents JAMA 2000 284 8 972 977 10.1001/jama.284.8.972 10944642

[B29] Shimada H Obuchi S Furuna T Suzuki T Nishizawa S Kojima M Risk factors of falls in the elderly people with dementia Gerontol Geriat Int 2003 3 S198

[B30] Shimada H Ota M Yabe N Obuchi S Furuna T Kojima M Suzuki T [Effect of predict falls for behavioral analysis in the elderly with dementia] Rigaku ryohogaku 2004 31 124 129

[B31] Shimada H Tiedemann A Lord SR Suzuki T The effect of enhanced supervision on fall rates in residential aged care Am J Phys Med Rehabili 2009 88 10 823 828 10.1097/PHM.0b013e3181b71ec2 21119315

[B32] Lord SR March LM Cameron ID Cumming RG Schwarz J Zochling J Chen JS Makaroff J Sitoh YY Lau TC Brnabic A Sambrook PN Differing risk factors for falls in nursing home and intermediate-care residents who can and cannot stand unaided J Am Geriatr Soc 2003 51 11 1645 1650 10.1046/j.1532-5415.2003.51518.x 14687397

[B33] Cummings SR Nevitt MC Kidd S Forgetting falls. The limited accuracy of recall of falls in the elderly J Am Geriatr Soc 1988 36 7 613 616 3385114 10.1111/j.1532-5415.1988.tb06155.x

